# Re-evaluation of NCGR Davis *Ficus carica* and *palmata* SSR profiles

**DOI:** 10.1371/journal.pone.0263715

**Published:** 2022-02-07

**Authors:** Richard Frost

**Affiliations:** Frost Concepts, Vista, California, United States of America; Sivas Bilim ve Teknoloji Universitesi, TURKEY

## Abstract

To date all public records of *F*. *carica* SSR profiles are from NCGR Davis. Prior studies of this data have not been received well because several of the stated relationships do not match what is observed in the field. Upon examination of the prior authors methods it is found that the 1979 Nei similarity measures are not valid distance metrics for the profiles thus invalidating their analysis of genetic distance. Further, the data are tensor in nature and it is shown here that "flattening the data" for use in a vector method will change the problem under study. Consequently the present analysis focuses on geometric, statistical, and biostatistical tensor-based methods–finding that only the latter produces results matching what is manually observed among the profiles. Combining this with historical breeding records and morphologic observations reveals that a modest portion of the profiled accessions are mislabeled–and also reveals the existence of previously undocumented close relations. Another area of concern in the prior studies is the statistical partitioning of the complete graph of distances to define clades. In the present analysis it is shown that genetic clades cannot be defined in this profile collection due to lack of cohesion in nearest neighbor components. It is also shown that it is presently intractable to significantly rectify gaps in the sample population by profile enrichment because the number of individuals in an entire population within the estimated profile distribution exceeds 10^14^. The profiles themselves are found to have very few occurrences of common values between the 15 loci and thus according to Fisher’s theory of epistatic variance no correlation to phenotype attributes is expected–a result verified by the original investigators. Therefore further discovery of appropriate markers is needed to fully capture geno- and pheno-type characteristics in *F*. *carica* and *F*. *palmata* SSR profiles.

## Introduction

Identifying plant varieties is an age-old human endeavor. Historically morphological traits were used to categorize specimens [1] into families, genera, species, and cultivars (for perennials: a plant selected from seedlings and asexually re-propagated for its desired characteristics). In the present day it is now possible to discern differences in plant cultivars via genetic measures. Some of these are “whole genetic sequence” while others focus on subsequences termed genetic profiles or “fingerprints”. One of these latter methods utilizes genetic profiles based on repeating values in the plant genome, termed SSR for “simple sequence repeats” [2]. So for example, if someone wishes to determine if two individual apple trees are the same cultivar, they can submit leaf samples to a plant ID lab and obtain an answer. In fact for some economically important crops, databases of SSR fingerprints have been established–so a plant ID lab can sometimes also determine which apple tree cultivar(s) the leaf specimens are from [3]. In addition to plant ID, those involved with plant breeding and germplasm repositories wish to determine the relationships among cultivars in a given collection, if not all cultivars worldwide [4]. For this application, a measure of “distance” between SSR profiles is needed, and it is helpful to have some reliable breeding records to establish ground truth and a scale of distance.

Genetic distance measures can be roughly classified into 4 categories: dynamic, statistical, geometric, and biostatistical. Dynamic methods use knowledge of linkage locations of loci along a genetic sequence to produce simulations of genetic crossovers in breeding, then analyze allele values to compute probabilities of relationships. Centimorgans are an example measure produced by dynamic simulation [5]. In contrast, static statistical and geometric measures do not require linkage data–which is a simplification in data acquisition, computation, and cost. Care however must be taken to determine which measures–if any, are relevant to the data. Statistical measures of genetic distance have their roots in comparing differences in populations, mostly originating in Fisher’s 1930 treatise on genetic variance [6]. Geometric measures use norm and norm-like measures to compute distances between spatial or spectral values. Biostatistical measures incorporate allele pattern matching to determine likely ancestral relations among cultivars and hybrids [7]. The technique of simple exclusion is one such approach applied to SSR profiles [8]. A similar method based on alleles patterns is introduced here.

Of interest in the present study are SSR profiles taken circa 2009 of the *Ficus carica* (fig) and *F*. *palmata* (Indian fig) collection at NCGR Davis [9]. The data are housed online at the USDA GRIN-Global site [10]. Structurally the profiles are 2×15 tensors of spatial data representing the total number of repeats of the dominant type per allele of 15 loci with 2 alleles each. An example is given in Table 1.

**Table 1:** Spatial SSR profile from NCGR 2010 for *F*. *carica* cultivar Kadota.

C22F1	C24H1	C26N1	C31F1	C35H1	C37N1	LM12H1	LM14H1	LM30N1	LM36N1	M1F1	M2H1	M3N1	M4F1	M8N1
283	272	234	224	254	204	214	200	243	248	172	153	120	194	171
283	272	234	239	254	208	243	200	245	248	189	167	132	218	175

Locus names are across the top. Values are the total number of repeats of the dominant type per allele (nomenclature: BP).

## Results and discussion

In the original published analyses of the NCGR profiles [9,11] the authors report using the 1979 Nei similarity measure [12]–i.e. Nei’s Eq. 8 or its isomorph Eq. 9 with proportion S given by Eq. 26. Both are vector in nature and thus inappropriate for the tensor data (see Methods section). Further, Eq. 8 fails metric requirements numerous times in the spatial and frequency domains of the SSR profiles (Table 2) and thus so will Eq. 9. All failures have magnitude of error equal or greater than the magnitude of minimum distance, and all were well above a numerical error tolerance of ε = 2×10^−9^.

**Table 2:** Metric test results of selected distance measures applied to 125 NCGR 2010 *Ficus sp*. SSR profiles.

δ	Type	Domain	Property Failed	Failures per Domain	Tests per Domain	Maximum |error|	Minimum |δ(a,b)|
Alleles Mask	tensor, biostatistical	spatial	none	n/a	n/a	n/a	n/a
Spectral 2	tensor, geometric	all	none	n/a	n/a	n/a	n/a
Nei	vector, geometric	spatial	4	10569	317750	10^−4^	10^−7^
Nei	vector, geometric	alleles frequencies	4	9145	317750	10^−1^	10^−6^
Nei	vector, geometric	loci frequencies	4	17428	317750	10^−1^	10^−5^
Nei	vector, geometric	population frequencies	4	10277	317750	10^−1^	10^−5^
Spectral Radius Angle	tensor, geometric	spatial	none	n/a	n/a	n/a	n/a
Spectral Radius Angle	tensor, geometric	alleles frequencies	4	2	317750	10^1^	10^0^
Spectral Radius Angle	tensor, geometric	loci frequencies	4	6	317750	10^1^	10^0^
Spectral Radius Angle	tensor, geometric	population frequencies	4	3	317750	10^1^	10^0^

The tensor measures Alleles Mask, Spectral 2, and Spectral Radius Angle were further analyzed for applicability to the profiles. The first is biostatistical and the others geometric. No static statistical tensor metric could be located. The computed distances were compared with measurements of allele similarities obtained from manual evaluation of the profiles. Results in the spatial domain edged out those in the alleles frequencies followed in turn by loci and population frequencies. However, neither geometric measure could completely resolve observed relations between all profiles–instead producing a few anomalies each due to their reliance on normative computations between numerical allele values (Tables 3 and 4). Hence the Alleles Mask metric is used for the remainder of the study.

**Table 3:** Distance partition key for selected metrics.

δ	Domain	Units	closest #1	closer #2	average #3	farther #4	farthest #5
Alleles Mask	spatial	Loci mismatches	[0., 2.)	[2., 3.5)	[3.5, 7.)	[7., 9.)	[9., 13.]
Spectral 2	alleles frequencies	BP frequencies	[0.008, 0.2)	[0.2, 0.54)	[0.54, 1.3)	[1.3, 1.5)	[1.5, 2.4]
Spectral Radius Angle	spatial	μradians	[0.594, 11.06)	[11.06, 21.514)	[21.514, 31.957)	[31.957, 42.387)	[42.387, 52.807]

**Table 4:** Example distances computed by 3 tensor measures, highlighting differences between geometric and pattern matching methods.

C1 DFIC, Label	C2 DFIC, Label	μr	BP *f*	Loci mismatches	Profile Analysis
7. Archipel	261. Encanto Brown Turkey	#1	#2	#1	14 ~ 0 ~ 0
102. Gulbun	126. Capri Q	#2	#3	#2	10 ~ 5 ~ 0
66. Kadota	20. Excel	#3	#3	#3	8 ~ 6 ~ 0
10. not Saleeb	205. LSU Hollier	#1	#4	#4	5 ~ 5 ~ 1
155. not California Brown Turkey	218. Fico Nero	#3	#4	#5	2 ~ 5 ~ 0

DFIC = accession #. Profile Analysis Key: (# exact loci matches) ~ (# single allele matches) ~ (# likely intra-loci crossovers).

**Fig 1:**
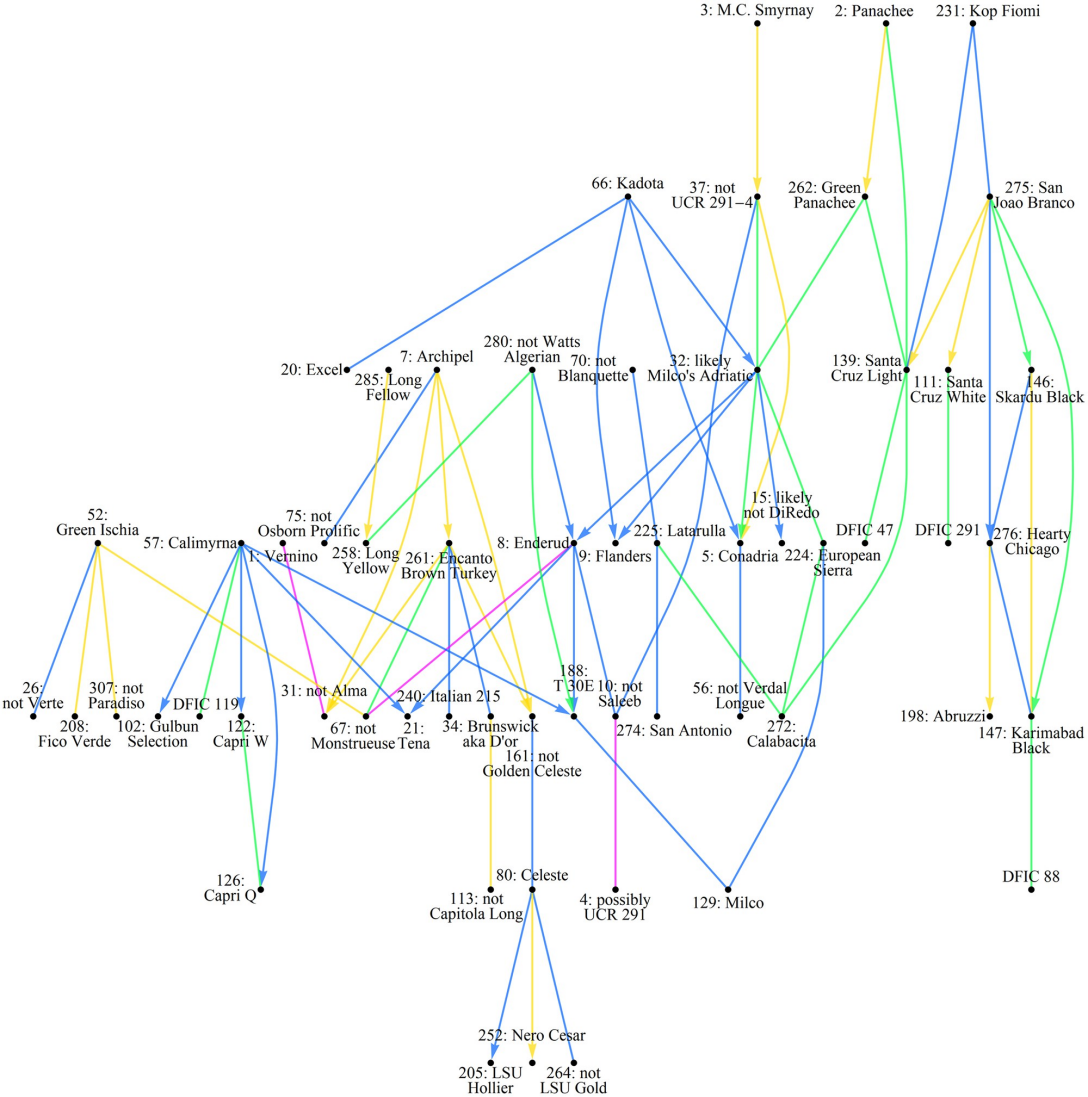
Determined genetic relations among NCGR accessions, part 1. Labels are only provided for verified accessions. Known/discovered descendants (if any) are denoted by arrows, not vertical hierarchy.

**Fig 2:**
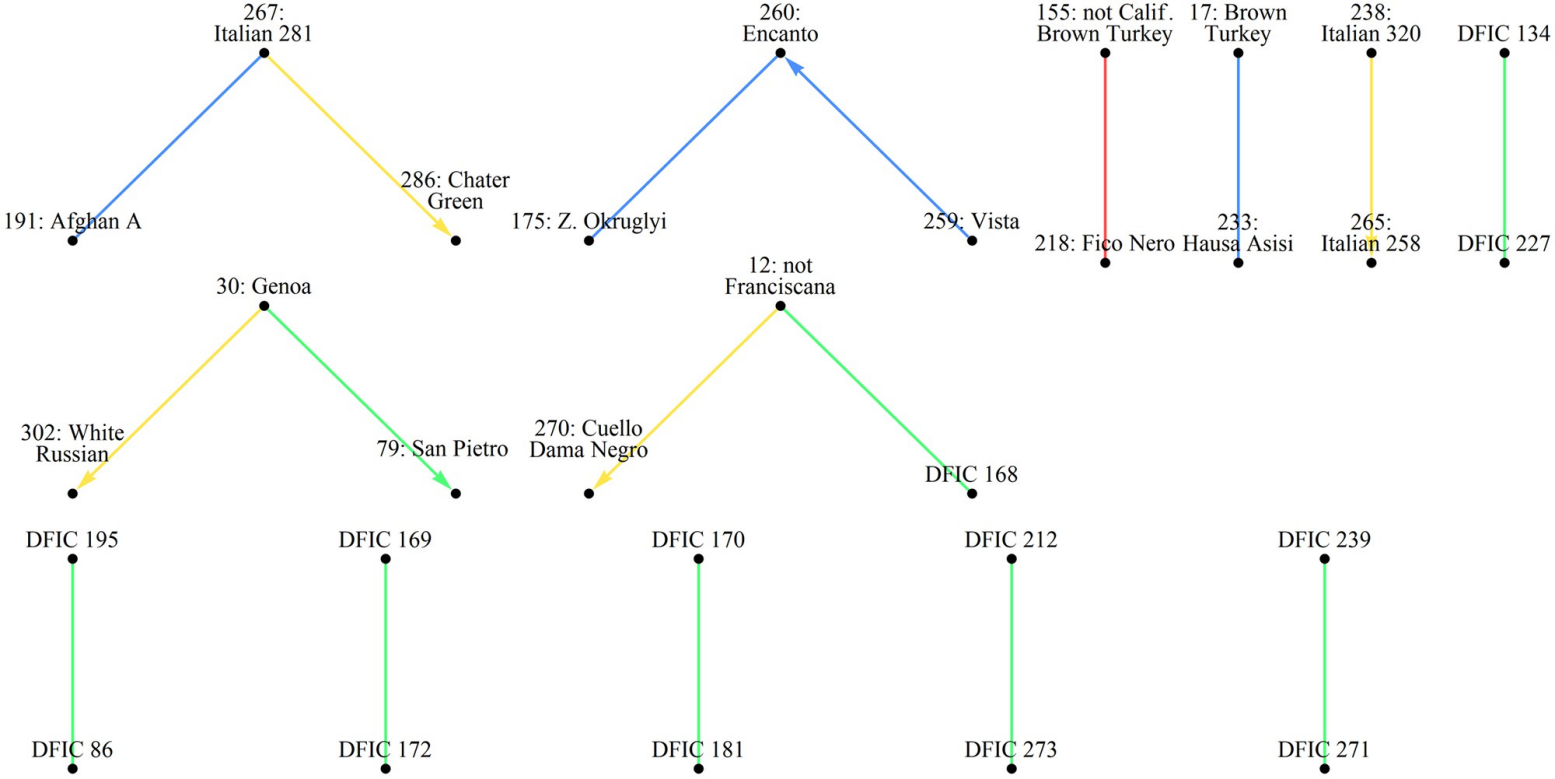
Determined genetic relations among NCGR accessions, part 2.

The Alleles Mask distances were then compared with breeding records documented by NCGR Davis (GRIN pedigree data), information from accession donor sites (GRIN passport data), and historical accounts [13] to determine label accuracy and ancestral relations among the NCGR *Ficus* collection. Seventeen were found incorrectly labeled, either due to being too distant according to breeding records or too close (sometimes identical!) to specimens documented as morphologically different. This is to be expected at a large repository with many donors over decades of operations without reliable means of authentication. As for ancestry, nine descendants listed in breeding records were identified but several more were also discovered including: Archipel → Encanto Brown Turkey, Genoa → San Pietro, Hearty Chicago → Abruzzi, Italian 281 → Chater Green, San Joao Branco → Santa Cruz White, and also San Joao Branco → Karimabad Black (repository accession DFIC 147). Although breeding records indicate that Excel is an offspring of Kadota, the profiles of these two demonstrate that the "Kadota" at NCGR Davis differs moderately from the parent of Excel. In addition, historical accounts and examination of profiles indicate that “Adriatic” (repository accession DFIC 32) is likely “Milco’s Adriatic” [13, p.407]. A graphic of these relations is provided in Figs 1 and 2.

The linear density of profiles within their esoteric space was estimated by computing *ρ*_*l*_ ≈ 0.733 the ratio of mean displacement đ to profiles perimeter radius r_p_ from central feature DFIC 32 Adriatic. For comparison, a “cannonball” packing of identical spheres in 3 dimensions has *ρ*_*l*_ ≈ 0.61 This demonstrates how dense SSR packings can be. In fact, any specific spatial profile in this set is distance 0 from the others in an average of 54.6% of alleles (identical allele value). Together these statistics demonstrate that the use of clustering techniques based on distance radii are inappropriate for this dataset. Nearest neighbor relations are necessary to overcome the high packing density. This is accomplished here by using Least Bridges Graphs as structural representations of profile relations.

**Fig 3:**
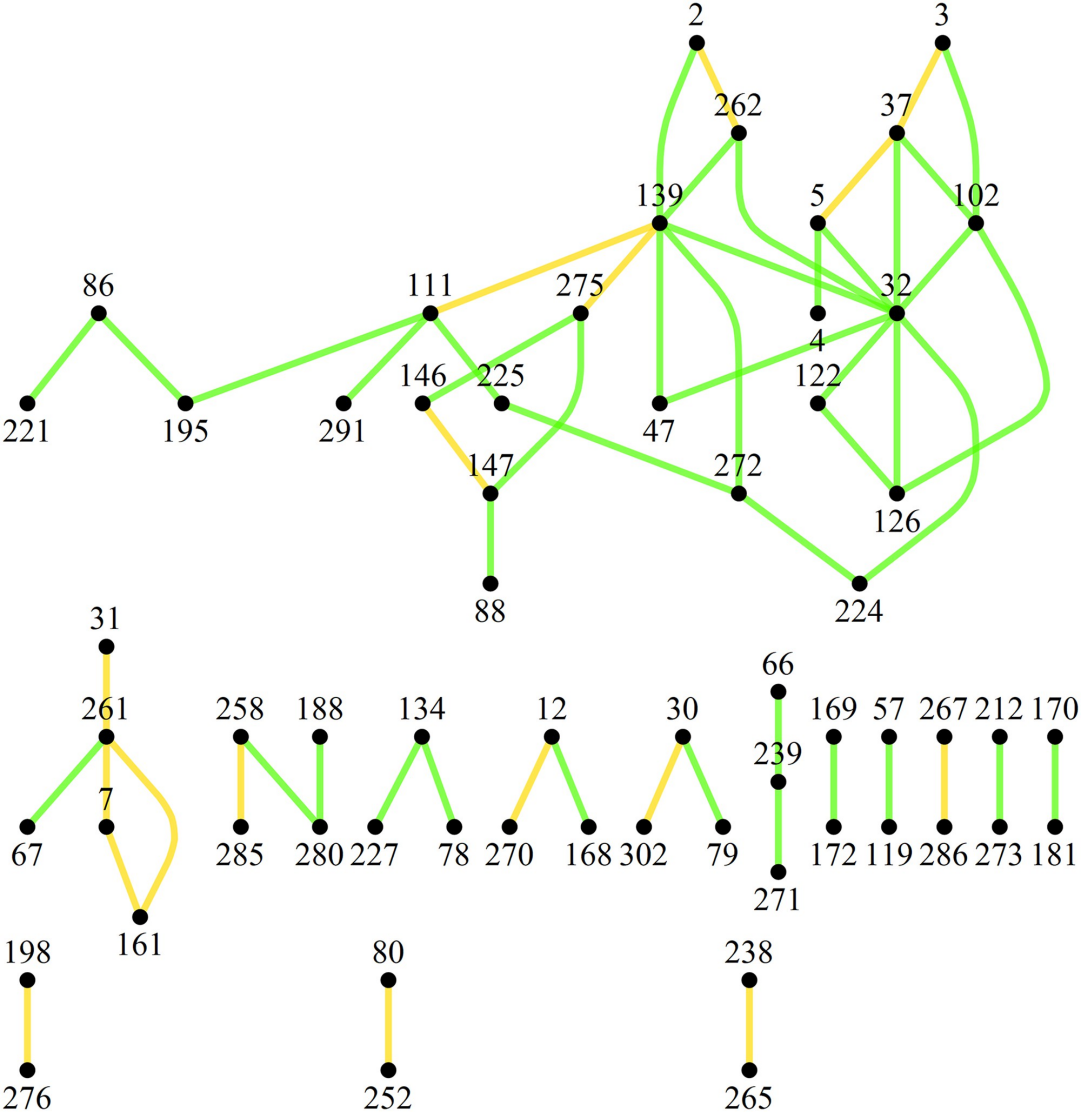
Components present in Least Bridges Graph when edge lengths are restricted to less than 3.5 Loci mismatches. Numbers refer to DFIC accessions.

The maximal Laplacian eigenvalue [14] λ_max_ ≈ 11.63 was computed for the connected Least Bridges Graph of distances. The maximal Laplacian is an upper bound on the number of edge frequencies and hence varieties of substructures within the graph. Organization of the SSR profiles into distance classes (hierarchies of nearest neighbor distances) demonstrates the infeasibility of large-scale biological clades that would span the collection. Specifically the lack of cohesion in the shortest distance classes prohibits larger scale aggregations of close relations. The result is that when the graph of connected components of profiles is restricted to using edge lengths with distance measure less than 3.5 Loci mismatches, no more than half of the profiles are used and the remaining are essentially cladeless. Also, most components constructed in this manner have the poor quality of containing 1 to 2 edges (Fig 3). Note that it is intractable to significantly rectify gaps in the sample population by profile enrichment because the number of individuals in an entire population within the estimated profile distribution exceeds 10^14^ (see Methods section). So although it is possible to apply partitioning software to the complete topological graph of the NCGR *F*. *carica* and *F*. *palmata* profiles, the majority of resulting clusters do not conform to expectations for biological clades.

An examination of frequencies of spatial values revealed only a few that occur in multiple loci (Table 5). If Fisher’s theory of epistatic variance [6] is correct then little correlation of these profiles with morphology data is expected–a result empirically determined in Aradhya’s study. Therefore a “whole genome” investigation of several cultivars will be necessary to determine a more exacting set of markers. If such an effort is undertaken it would also be helpful to secure Loci to identify the various odd sexual states of *Ficus carica*.

**Table 5:** Spatial values found in multiple loci.

C22F1	C24H1	C26N1	C31F1	C35H1	C37N1	LM12H1	LM14H1	LM30N1	LM36N1	M1F1	M2H1	M3N1	M4F1	M8N1
					200 210	214 233 243	200 210	233 243		155	155		214	

## Methods

### Vectors vs. tensors

Few purely tensor genetic distance measures exist in the literature. As such it is a common but dubious practice among practitioners to “flatten” tensors (string out in single vector) for use in vector measures. Consider for a moment though the p×q non-trivial tensors A, B, A ≠ B, and C = B—A, which in our Euclidean minds we would like to think of as edges of "triangle" A,B,C. Now compute the angle

$$\text{cos}\theta \left(A,B\right)={\left\|A\cdot B^{T}\right\|}_{2}/\left({\left\|A\right\|}_{2}\cdot {\left\|B\right\|}_{2}\right)$$

which we would like to think of as opposite of C. But since perpendicularity (a restricted form of orthogonality) is ill-defined with tensors, we discover that the law of cosines almost always fails for our "triangle" because it depends on a non-existent “edge” perpendicular to B:

$${\left\|C\right\|}^{2}\neq {\left\|A\right\|}_{2}^{2}-2\cdot {\left\|A\cdot B^{T}\right\|}_{2}+{\left\|B\right\|}_{2}^{2}.$$

To make matters worse, we also discover that with few exceptions:

$$\theta \left(A,B\right)+\theta \left(B,C\right)+\theta \left(C,A\right)\neq \pi .$$

Hence tensors are different from vectors and “flattening” tensors into vectors changes the problem under study. Further: any values computed by nontrivial δ in the vector space are useless because an inverse to translate them back to corresponding δ values in the original tensor space is infeasible due to the nature of the projection. Consequently the practice of flattening tensors for the purpose of vector computation should be avoided.

### Comparable distances

The values produced by a distance measure δ are not considered valid for comparison unless δ is a qualified metric [15]. For the general case of a complete directed graph this means:

1. δ(a, a) = 0 for every profile a
2. δ(a, b) > 0 for all profiles a with single edge path to b and a ≠ b
3. δ(a, b) = δ(b, a) for all single undirected edges between profiles a, b
4. δ(a, c) ≤ δ(a, b) + δ(b, c) for all profiles a, b, c having single edge path a to b, b to c, and a to c.

Some measures come “pre-proven” for undirected graphs, e.g. Euclidean. Having a pre-proven measure does not mean that numerical instability or ill-conditioning [16] will not cause your data to fail. The Mahalanobis measure is a prime example of where this can occur.

### Tensor metrics

#### Alleles mask

**Fig 4:**
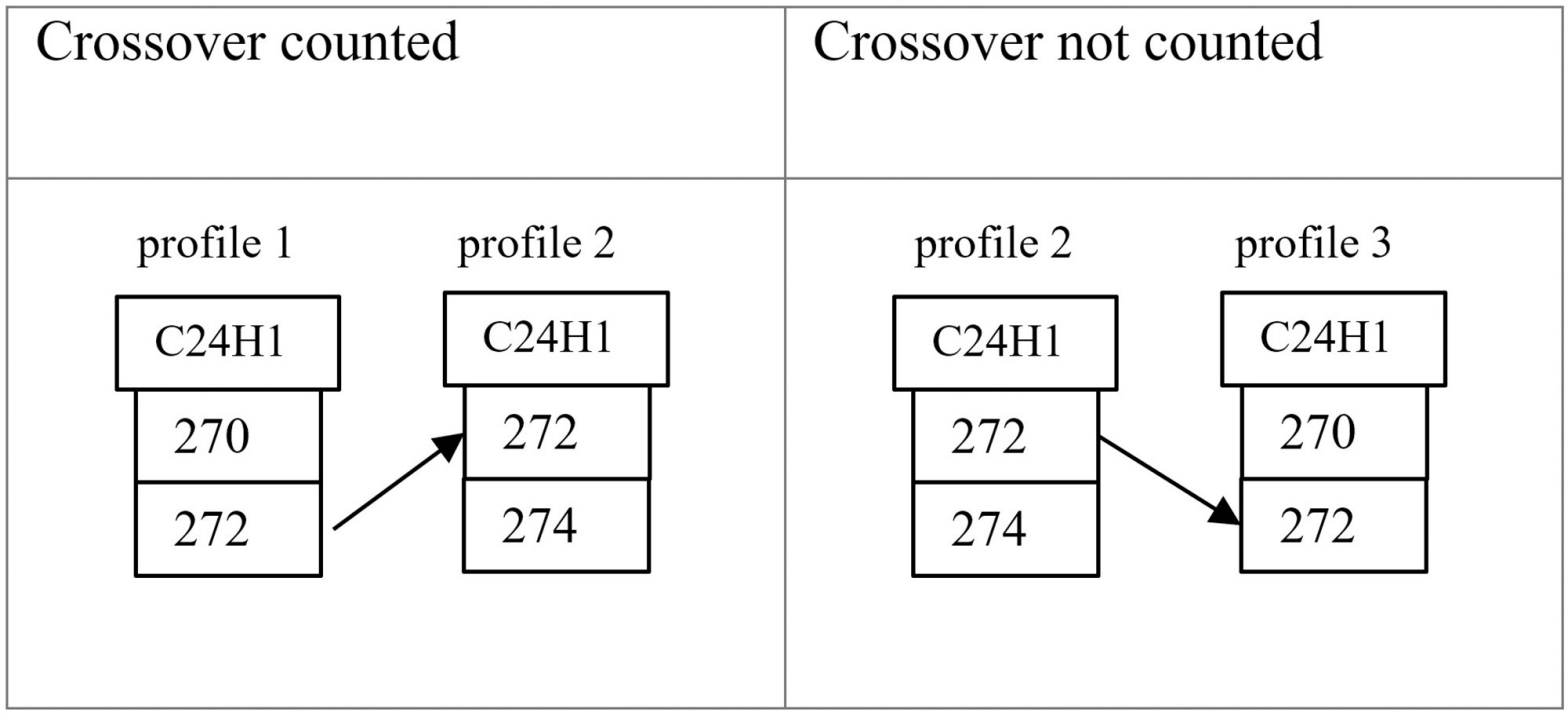
Counting of common allele values. The back allele of C24H1 commonly has the value 272.

A measure with units of Loci mismatches. Denote F_i_(A_n_, A_m_) = a full Loci match between profiles A_n_ and A_m_ at locus i. Likewise denote S_i,j_(A_n_, A_m_) = a single allele match at allele j of locus i in A_n_ and A_m_, but not “double counting” those in full Loci matches. And finally denote C_i,j_(A_n_, A_m_) = an intra-loci crossover match from allele j to allele ~j of locus i in A_n_ and A_m_−but not double counting those from full Loci matches of identical values, and also not counting those where the target allele is one of the high frequency (e.g. ≥ 84%) values in the sample population (Fig 4). Note that this criteria can cause C_i,j_(A_n_, A_m_)≠ C_i,−j_(A_m_, A_n_), thus producing a directed graph.

To compute, begin with a profile mask containing all 1’s:

$$\begin{array}{ccccccccccccccc} 1 & 1 & 1 & 1 & 1 & 1 & 1 & 1 & 1 & 1 & 1 & 1 & 1 & 1 & 1\\ 1 & 1 & 1 & 1 & 1 & 1 & 1 & 1 & 1 & 1 & 1 & 1 & 1 & 1 & 1 \end{array}$$

then set locations to 0 wherever F, S, or C occur and divide the total by 2 to produce units of Loci mismatches.

For example, consider the profile of DFIC 66 Kadota:

$$\begin{array}{ccccccccccccccc} 283 & 272 & 234 & 224 & 254 & 204 & 214 & 200 & 243 & 248 & 172 & 153 & 120 & 194 & 171\\ 283 & 272 & 234 & 239 & 254 & 208 & 243 & 200 & 245 & 248 & 189 & 167 & 132 & 218 & 175 \end{array}$$

and the profile of DFIC 20 Excel:

$$\begin{array}{ccccccccccccccc} 283 & 272 & 234 & 224 & 254 & 204 & 214 & 200 & 243 & 248 & 189 & 153 & 124 & 218 & 171\\ 285 & 272 & 234 & 239 & 254 & 208 & 214 & 200 & 247 & 248 & 189 & 167 & 132 & 218 & 171 \end{array}$$

The mask produced for the distance Kadota to Excel is

$$\begin{array}{ccccccccccccccc} 0 & 0 & 0 & 0 & 0 & 0 & 0 & 0 & 0 & 0 & 1 & 0 & 1 & 1 & 1\\ 1 & 0 & 0 & 0 & 0 & 0 & 1 & 0 & 1 & 0 & 0 & 0 & 0 & 0 & 1 \end{array}$$

with computed distance 4.0 Loci mismatches.

#### Spectral 2

Here, the spectral radius [17] order 2 tensor norm for positive rational values

$$\rho (A)\equiv \underset{i\in {\mathbb{N}}_{p}}{\text{max}}\left|\sqrt{\lambda_{i}\left(A\cdot A^{T}\right)}\right|,\left(A\in {\mathbb{Q}}_{p\times q}^{+}\right)\wedge \left(p\leq q\right)$$

is used to induce a Euclidean-like metric for tensors

$$S2(A_{n},A_{m})\equiv \rho \left(A_{n}-A_{m}\right).$$

#### Spectral Radius Angle

In this application of the spectral radius norm a spatial tensor metric with units of radians is obtained

$$SRA\left(A_{n},A_{m}\right)\equiv {\text{cos}}^{-1}\left[\rho \left(A_{n}\cdot A_{m}{}^{T}\right)/\left[\rho \left(A_{n}\right)\cdot \rho \left(A_{m}\right)\right]\right].$$

### Breeding records

**Fig 5:**
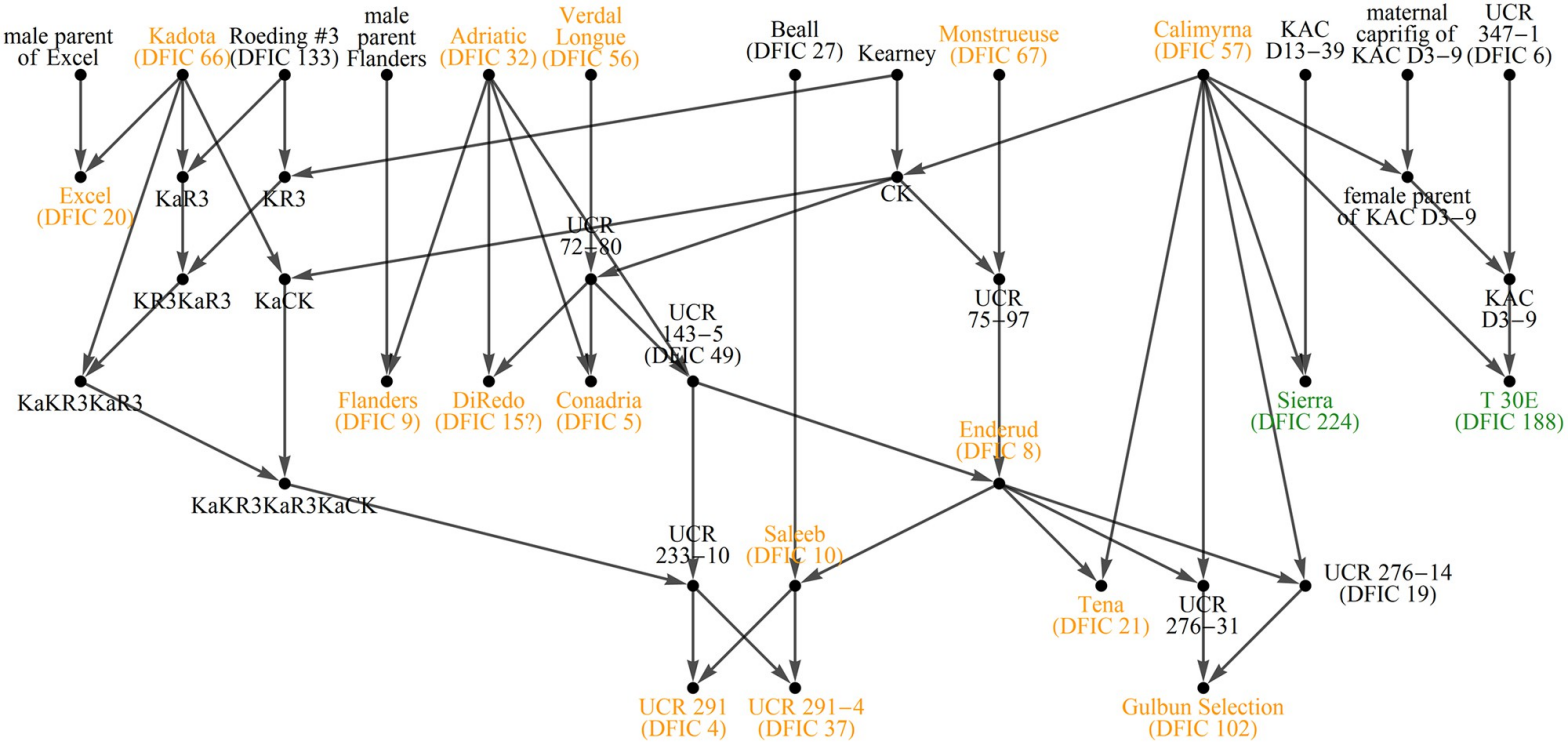
Diagrams of breeding records for fig accessions at NCGR Davis, part 1. Arrows point to progeny. Shaded names are NCGR accessions with matching labels and genetic data. Some labels were found to be inaccurate after genetic profile analysis. Color indicates breeding location. Gold = UC Riverside, Green = Kearney Ag Center, Purple = Louisiana State University, Maroon = Texas A&M University.

**Fig 6:**
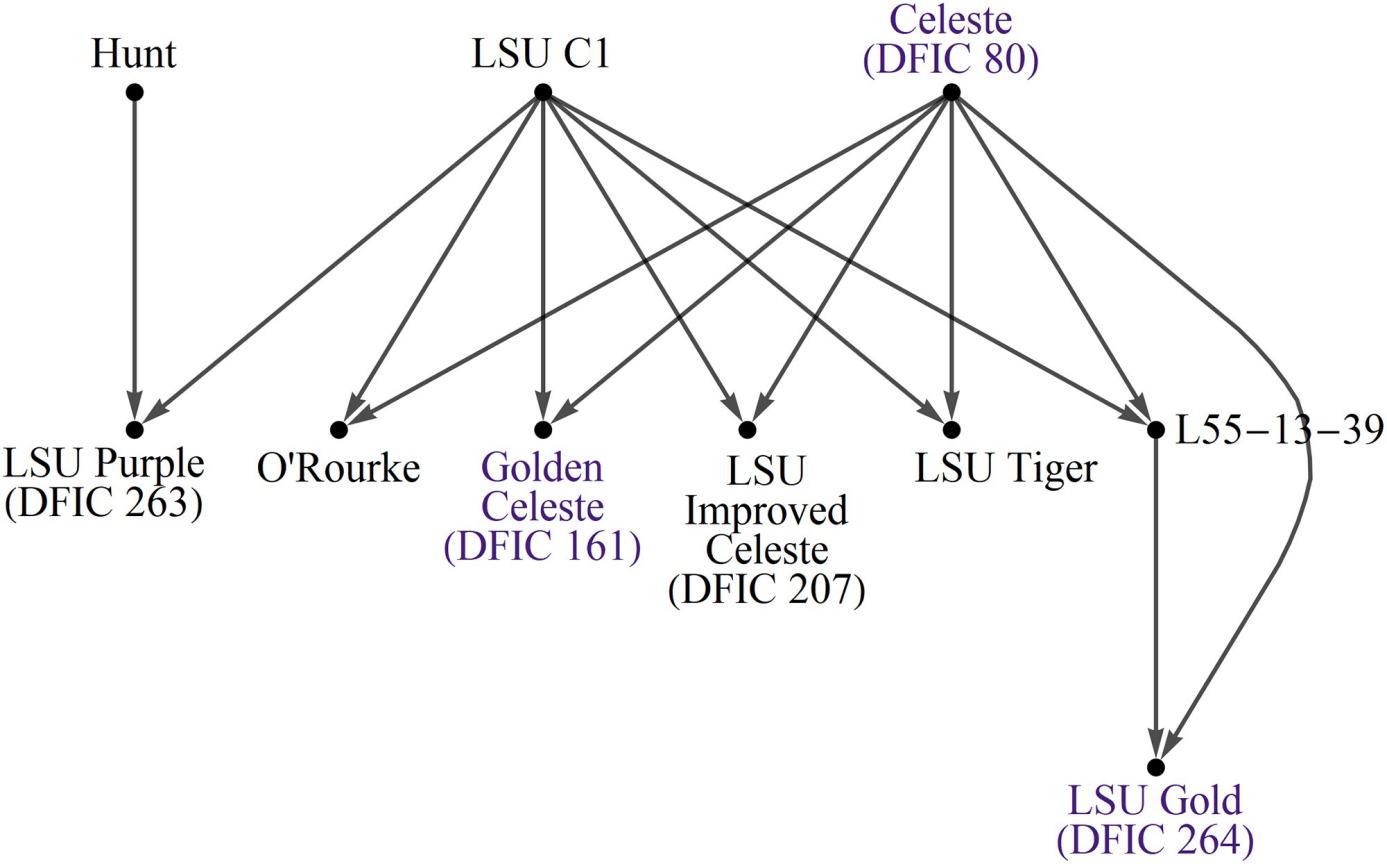
Diagrams of breeding records for fig accessions at NCGR Davis, part 2.

**Fig 7:**
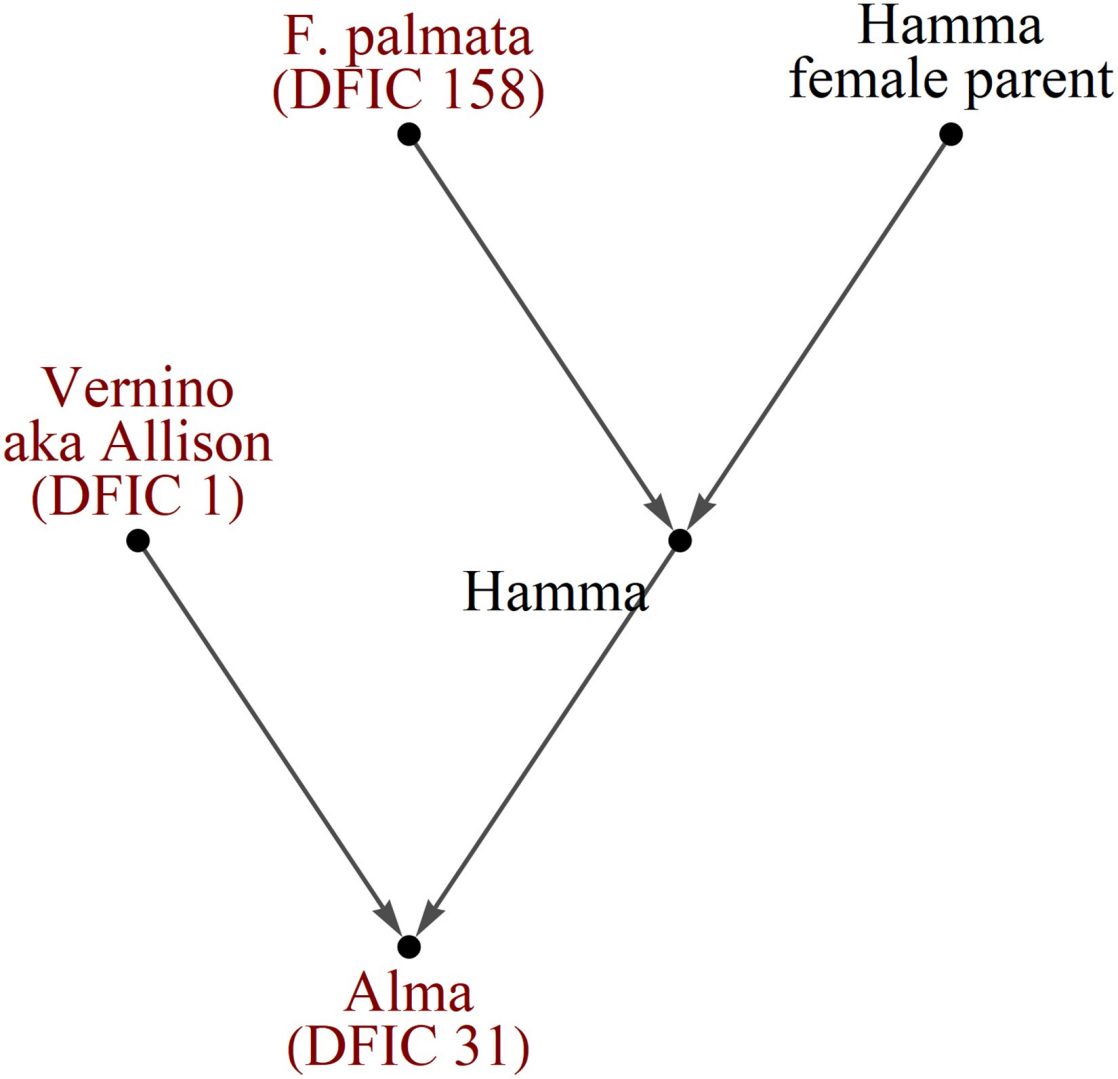
Diagrams of breeding records for fig accessions at NCGR Davis, part 3.

Also available at the USDA GRIN-Global site are breeding records for *Ficus sp*. from historical USDA and UC breeding programs. These were downloaded and assembled into the diagrams of Figs 5–7. The records were used to guide manual side-by-side profile analysis along with comparisons of distance measure results presented in this article.

### Historical accounts

Ira Condit’s voluminous monograph of fig varieties was published by the UC research periodical Hilgardia in 1955 [13]. It is in some respects a massive review of the fig variety literature, spanning centuries of publications. It also contains Condit’s own anecdotes and observations, plus those of his predecessor Gustav Eisen. Although Condit’s analysis of cultivar synonyms is based on the questionable practice of morphology ID, the historical phenotype observations are useful for ferreting out what a cultivar is not. For example, Condit (and others) considered the Osborn Prolific cultivar to be identical to Archipel [13, p.414]. However, the SSR profiles of accessions with those labels in the NCGR collection are not similar–placing the labels of those accessions in question. Alternately there is historical precedence that California Brown Turkey (aka San Piero) is different from a cultivar named Brown Turkey imported from Europe [13, p.428]. But the genetic profiles of the accessions with these labels at NCGR Davis are identical–putting the accuracy of those two labels in question as well. Several discrepancies were discovered in this manner, leading to a puzzle of question marks. Many of these were half-way resolved (one of the pair but not the other) with the aid of breeding records and matching profile comparisons. A few were taken on faith in the passport data of the accession–including Archipel. Questionable accessions that could not be resolved are among those labeled “not” in Figs 1 and 2.

### Least Bridges Graph construction

**Fig 8:**
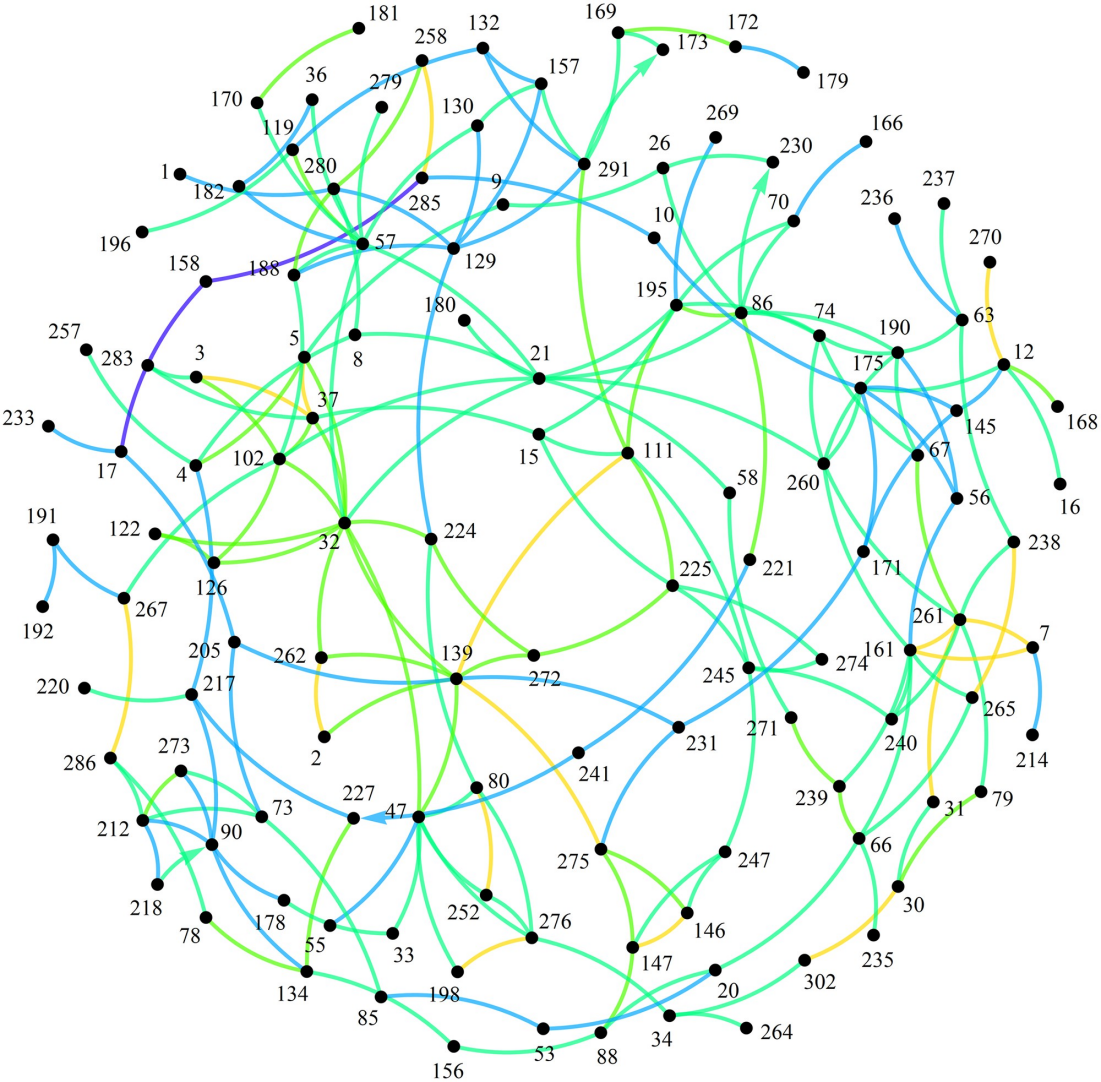
Connected Least Bridges Graph produced by Alleles Mask metric. Distance class hues = {∎, ∎, ∎, ∎, ∎, ∎, ∎}, distance class partitions (loci mismatches) = {0.5, 2., 3.5, 4.5, 6., 7.5, 8.5, 13.}. Numbers refer to DFIC accessions. Arrowheads denote directed edges produced by the Alleles Mask metric.

Least Bridges Graphs are a method of visualizing nearest-neighbor relationships in abstract spaces. They are constructed by first considering the vertices (e.g. genetic profiles) as disconnected components, then incrementally adding the shortest available edge connection (i.e. edge representing distance between the two components). Edges are only added between disconnected components and thus termed "bridges" [14]. A new component is created each time an edge is added, replacing the prior two. If there are multiple edges of the same distance that qualify then the entire set is added, possibly engulfing multiple components. This latter requirement ensures edge multiplicity is not ignored–an error in many cluster algorithms used for distances (e.g. the underlying k-neighbors function in Mathematica^®^ v12.1 NearestNeighborGraph [18] for small k). The distances among components must be re-evaluated after an edge or edge set is added. Inter-component distances are determined by selecting the shortest vertex-to-vertex distance between them. The process is continued recursively until a prescribed limit is reached (e.g. a maximum distance) or a connected graph is achieved. Having only 26 unique distances within this dataset, the Alleles Mask metric produces a connected graph in 14 iterations (Fig 8). In contrast, all distances produced by the Spectral Radius Angle metric were unique and required 1866 iterations to achieve a connected graph.

### The intractability of profile enrichment

The fig collection from NCGR Davis is not a random sample of individuals from the worldwide population, but mostly a selection of preferred cultivars from commercial production and private collections [19]. As such the allele frequency distribution is somewhat representative of “desirable” figs. A reasonable question is: what is the largest possible collection of these “desirable” profiles having the same distribution?

As a first estimate consider the product of the number of unique alleles values per loci

C=8,709,120,000

This assumes the frequencies are accurate with no multiplicities. To include multiplicity and uncertainty in the estimate, introduce a 2% frequency variation in numerator values that conserves probability so the sum of frequencies per loci still adds to 1. In particular, numerators of alleles frequencies of each loci are permitted to vary by {-2,-1,0,+1+,2} provided the sum of the frequencies per loci adds to 1. (The denominator is held constant at N = 125.) Now check the numerators per loci and count the greatest common divisors. Selecting the min, median, and max produces

$$C=2.85\times 10^{14}\left(\text{min}\right),4.18\times 10^{22}\left(\text{median}\right),3.16\times 10^{30}\left(\text{max}\right).$$

The purpose of going to this trouble is to demonstrate that sample sizes of 1000, 2000, or even 200000 are insignificant [20] when compared to the vast number of possibilities that occur for this distribution. If the goal of a profile enrichment exercise is to fill in gaps between nearest-neighbor components then at least 10^10^ (more likely 10^18^) profiles will be needed for a statistically significant sample. This is an intractable situation unless someone can express it as a satisfiability problem for quantum computing.
